# Progressive associative phonagnosia: A neuropsychological analysis

**DOI:** 10.1016/j.neuropsychologia.2009.12.011

**Published:** 2010-03

**Authors:** Julia C. Hailstone, Sebastian J. Crutch, Martin D. Vestergaard, Roy D. Patterson, Jason D. Warren

**Affiliations:** a Dementia Research Centre, Institute of Neurology, University College London, Queen Square, London WC1N 3BG, United Kingdom; b Centre for the Neural Basis of Hearing, Physiology Department, University of Cambridge, Cambridge, United Kingdom

**Keywords:** Voice, Face, Person knowledge, Prosopagnosia, Frontotemporal lobar degeneration, Dementia

## Abstract

There are few detailed studies of impaired voice recognition, or phonagnosia. Here we describe two patients with progressive phonagnosia in the context of frontotemporal lobar degeneration. Patient QR presented with behavioural decline and increasing difficulty recognising familiar voices, while patient KL presented with progressive prosopagnosia. In a series of neuropsychological experiments we assessed the ability of QR and KL to recognise and judge the familiarity of voices, faces and proper names, to recognise vocal emotions, to perceive and discriminate voices, and to recognise environmental sounds and musical instruments. The patients were assessed in relation to a group of healthy age-matched control subjects. QR exhibited severe impairments of voice identification and familiarity judgments with relatively preserved recognition of difficulty-matched faces and environmental sounds; recognition of musical instruments was impaired, though better than recognition of voices. In contrast, patient KL exhibited severe impairments of both voice and face recognition, with relatively preserved recognition of musical instruments and environmental sounds. Both patients demonstrated preserved ability to analyse perceptual properties of voices and to recognise vocal emotions. The voice processing deficit in both patients could be characterised as associative phonagnosia: in the case of QR, this was relatively selective for voices, while in the case of KL, there was evidence for a multimodal impairment of person knowledge. The findings have implications for current cognitive models of voice recognition.

## 1 Introduction

Prosopagnosia, or impaired recognition of familiar faces, has been widely studied both in patients with acquired brain lesions and as a developmental disorder (Barton, 2003; De Renzi, Faglioni, Grossi, & Nichelli, 1991; Duchaine & Nakayama, 2006; Gainotti, 2007b; Lucchelli & Spinnler, 2008; Young, Newcombe, de Haan, Small, & Hay, 1993). A striking clinical illustration of the acquired breakdown of face recognition ability is the syndrome of progressive prosopagnosia, a canonical manifestation of right temporal lobe atrophy in the frontotemporal lobar degeneration (FTLD) spectrum (Chan et al., 2009; Evans, Heggs, Antoun, & Hodges, 1995; Josephs et al., 2008; Joubert et al., 2003, 2004; Tyrrell, Warrington, Frackowiak, & Rossor, 1990). Progressive prosopagnosia may represent a variant of semantic dementia dominated by deficits of nonverbal knowledge, including knowledge of familiar people (Gainotti, 2007a; Gainotti, Ferraccioli, Quaranta, & Marra, 2008; Gentileschi, Sperber, & Spinnler, 1999; Gentileschi, Sperber, & Spinnler, 2001; Hanley, Young, & Pearson, 1989; Snowden, Thompson, & Neary, 2004; Thompson et al., 2004). The syndrome is of considerable neuropsychological as well as clinical importance because it provides a window on the organisation of person knowledge in the brain (Bruce & Young, 1986; Burton & Bruce, 1993; Lucchelli & Spinnler, 2008; Lyons, Kay, Hanley, & Haslam, 2006; Snowden et al., 2004; Thompson et al., 2004; Warrington, 1979). However, faces, while typically the most salient source of nonverbal information about other people, are only one of several components of person knowledge. Other channels of person knowledge, notably voices, commonly become affected with evolution of the progressive prosopagnosia syndrome (Gainotti, Barbier, & Marra, 2003; Gainotti et al., 2008; Gentileschi et al., 2001), however selective impairment of voice processing, or phonagnosia, is less commonly reported. Phonagnosia has been described as a developmental disorder (Garrido et al., 2009) and, more commonly, in association with focal damage involving the right or left temporal lobe or the right parietal lobe (Ellis, Young, & Critchley, 1989; Hanley et al., 1989; Lang, Kneidl, Hielscher-Fastabend, & Heckmann, 2009; Neuner & Schweinberger, 2000; Van Lancker & Canter, 1982; Van Lancker & Kreiman, 1987; Van Lancker, Cummings, Kreiman, & Dobkin, 1988; Van Lancker, Kreiman, & Cummings, 1989), consistent with the distributed network of areas engaged by voice processing tasks in functional imaging studies of healthy subjects (Belin, Zatorre, & Ahad, 2002; Imaizumi et al., 1997; Nakamura et al., 2001; von Kriegstein, Kleinschmidt, & Giraud, 2006). However, phonagnosia is much less well characterised than prosopagnosia; indeed, voice processing is often anecdotally assumed to be normal in early progressive cases (Evans et al., 1995; Gentileschi et al., 1999; Joubert et al., 2003), and is generally assessed only following the development of face recognition deficits (Gainotti et al., 2003; Gentileschi et al., 1999, 2001) and may not be identified as a clinical issue. Aside from the technical difficulty of assessing voice processing in clinical settings, this may be because phonagnosia is intrinsically less salient than face or name recognition deficits (Neuner & Schweinberger, 2000). Nevertheless, phonagnosia may be a significant and disabling clinical issue, especially in situations where compensatory cues are reduced or unavailable (e.g., over the telephone).

While cognitive neuropsychological models of person identification have been developed chiefly for the case of faces, such models provide a framework for analysing the processing of voices and the effects of disease. Current models of person identification have been heavily influenced by data on face processing (Bruce & Young, 1986; Ellis, Jones, & Mosdell, 1997; Lucchelli & Spinnler, 2008; Neuner & Schweinberger, 2000). These models agree broadly on the segregation of perceptual processing (via parallel processing of faces, voices and name stimuli), and a hierarchical processing of person information from early perceptual to higher semantic levels of processing. However, the detailed predictions of these models and their neuropsychological instantiation have yet to be fully worked out. Elaborations of the Bruce and Young model applied specifically to voice processing have been proposed (Belin, Zatorre, Lafaille, Ahad, & Pike, 2000; Belin, Fecteau, & Bedard, 2004; von Kriegstein, Kleinschmidt, Sterzer, & Giraud, 2005; von Kriegstein et al., 2006), but have rarely been systematically assessed in brain-damaged populations. Key unresolved issues include the degree of modality-specificity of face and voice processing deficits; the level at which any modality specificity arises; the extent to which perceptual and semantic levels of processing are interdependent; and the status of voices versus other categories of auditory objects, and other fine-grained semantic categories beyond the domain of person knowledge.

We had the opportunity to address these issues in a case control study of two patients with deficits of person knowledge in the context of FTLD. The index case, patient QR, exhibited progressive loss of recognition of familiar voices as a leading clinical symptom, while the second patient, KL, presented with progressive prosopagnosia without a clinical complaint of altered voice recognition. We designed a series of neuropsychological experiments to characterise in detail different levels of voice processing in both patients. Perceptual and semantic processing of voices was assessed in relation to processing of faces, recognition of vocal emotions and identification of non-vocal sounds, in order to assess the modality- and material-specificity of any voice processing deficit.

## 2 Methods and results

### 2.1 Subject details

#### 2.1.1 Patient QR

**Fig. 1:**
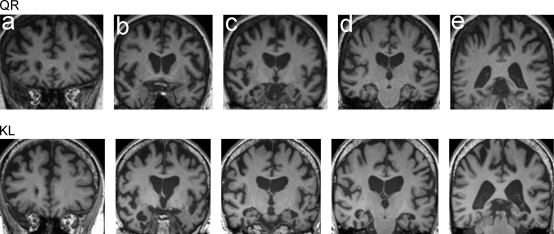
Representative T1-weighted coronal brain MRI sections from each patient (the right hemisphere is shown on the left side of each image). Sections have been selected to show the following regions of potential relevance to voice processing deficits: a, frontal lobes; b, temporal poles; c, anterior temporal lobes; d, mid-temporal lobes including Heschl's gyri; e, temporo-parietal junction. Focal cerebral atrophy is shown in both patients: in QR, bilateral fronto-temporal atrophy accentuated in the right anterior temporal lobe and extending posteriorly and including the superior temporal sulcus; and in KL, bilateral predominantly anterior temporal lobe atrophy, more marked on the right side and in the inferior temporal cortices including the fusiform gyrus.

This 61-year-old right-handed female hairdresser presented with a 2-year history of insidiously progressive behavioural decline, with impassivity, obsessionality, clock-watching, loss of empathy and development of a sweet tooth. Impaired voice recognition was an early symptom. When first assessed she was no longer able to identify the voices of her children on the telephone, nor did she evince any sense that their voices were familiar. In contrast, recognition of faces had not been similarly affected: she consistently recognised family members, and despite the suggestion of some recent difficulty in identifying friends in social situations, she continued to exhibit a sense of familiarity toward them. On examination there was evidence of executive dysfunction, disinhibition, perseveration and impulsivity. Naming and verbal memory were impaired whereas early visual perceptual skills were preserved. The general neurological examination was unremarkable. Peripheral hearing assessed using pure tone audiometry was within normal limits for age. Brain MRI (Fig. 1) showed bilateral fronto-temporal atrophy somewhat accentuated in the right anterior temporal lobe but extending posteriorly within the temporal lobe and including the superior temporal sulcus, with no significant cerebrovascular changes. The clinical diagnosis was behavioural variant frontotemporal dementia.

#### 2.1.2 Patient KL

This 72-year-old left-handed male academic presented with an 8-year history of insidious cognitive decline; initially reporting a difficulty in recognising neighbours and other close acquaintances, followed by progressive difficulties with word finding and topographical memory and mild behavioural changes. He had been born in the US but had lived in the UK periodically for over 50 years and consistently for the last 11 years. There was no history to suggest phonagnosia though he reported that he found understanding unfamiliar accents increasingly difficult. On examination there was evidence of mild disinhibition and impaired recognition of famous faces, with preservation of early visual perceptual skills. The general neurological examination was unremarkable. Peripheral hearing assessed using pure tone audiometry was within normal limits for age. Brain MRI (Fig. 1) showed bilateral predominantly anterior temporal lobe atrophy, more marked on the right side and in the inferior temporal cortices including the fusiform gyrus. The clinical diagnosis was temporal variant frontotemporal lobar degeneration with progressive right temporal lobe atrophy.

#### 2.1.3 Healthy controls

The experimental tasks were administered to a control group of healthy older individuals. All were native English speakers and British residents with no history of neurological or psychiatric illness and had normal screening audiometry. Perceptual and semantic tasks were administered to 24 control subjects (17 females; mean age = 64.5, SD = 4.3, range: 55–73; mean years of education 15.5, SD = 3.5, range: 11–25): between 20 and 24 controls completed each of the voice processing tests, and 15 controls also completed a test of environmental sound recognition. In addition, a test of vocal emotion recognition was administered to a separate group of 22 older controls (12 females; mean age = 67.2, SD = 8.8, range: 53–78).

The background media exposure of the control subjects and both patients was assessed formally as a potentially relevant factor influencing voice recognition ability. The procedure and results are summarised in Appendices A and B.

The study was approved by the local institutional research ethics committee and all subjects gave informed consent in accord with the principles of the Declaration of Helsinki.

### 2.2 Background neuropsychological assessment

The performances of QR, KL and healthy controls on general neuropsychological tests and standard tests assessing identification of faces and topographical landmarks, examples of ‘unique entities’ in the visual modality, are summarised in Table 1. Both QR and KL had evidence of anomia on the Graded Naming Test (GNT) (Warrington, 1997), and QR had evidence of additional impairments of single word comprehension (abstract synonyms: (Warrington, McKenna, & Orpwood, 1998)), surface dyslexia on the National Adult Reading Test (NART) (Nelson, 1982) and executive function (Delis, Kaplan, & Kramer, 2001); neither patient showed a deficit of short term memory or early visual perceptual function. On the standard Famous Faces Test (Warrington & James, 1967) QR performed at the 5th percentile on face naming and normally on the face recognition component of the test, while KL showed impairments on both tasks. On a test assessing naming and recognition of 15 famous London landmarks from photographs (Whiteley & Warrington, 1978), QR and KL each performed below the 5th percentile for naming and at the 5th percentile for recognition.

### 2.3 Experimental investigations: structure and general procedure

Voice recognition was assessed using tests of familiarity and identification of famous voices. Normal voice recognition ability was quantified in a pilot study (see Appendix B) in healthy older adult controls, as it has been shown previously that normal individuals have more difficulty recognising public figures from voice than from faces or name (Damjanovic & Hanley, 2007; Ellis et al., 1997; Hanley, Smith, & Hadfield, 1998). The specificity of any voice recognition deficit was assessed using recognition of public figures represented in other modalities (faces and names), and within the auditory modality using tests of vocal emotion recognition and environmental sound identification. In order to assess the effects of perceptual factors on any voice recognition deficit, discrimination of unfamiliar voices and perceptual analysis of vocal properties (speaker size and gender) were investigated in separate experiments. Single-case results were assessed in relation to the control sample using the method of Crawford and Howell (1998).

Tests were administered in divided sessions. In order to minimise priming of voice recognition from other modalities, recognition tests were administered in a fixed order, motivated by evidence from the pilot control study (Appendix B) that the public figures selected were all better recognised from face than from voice. Voice familiarity, naming and identification tasks were performed first, followed by face familiarity, naming and identification tasks, and finally the name familiarity task; the order of stimuli was randomised within each modality. Voice stimuli were delivered from digital wavefiles via a personal computer in free-field at a comfortable constant listening level. In cross-modal matching tasks, names were presented simultaneously spoken and written. Stimuli were presented in randomised order. For each test, the experimenter first ensured that subjects understood the task; however no feedback was given during the test proper.

### 2.4 Experiment 1: familiarity of voices, faces and personal names

The aim of this experiment was to assess the familiarity of famous voices for QR and KL, and to compare this with familiarity judgments for faces and names of the same individuals. Voice samples were selected based on the initial pilot study in a separate group of healthy controls, and face photographs for the same individuals were used for the face recognition task. The final set of comprised 24 British and American public figures: (see Appendix C) 10 politicians, five actors, seven media personalities from television and radio, and two members of royalty. Examples of stimuli are available from the authors.

Voice samples and photographs were chosen so as to minimise other potential semantic cues to recognition. A selection of 24 unfamiliar voices and faces that were classified as unfamiliar by >75% of controls were included in the final test; these were matched by gender to the familiar set and approximately matched for age and accent. Unfamiliar personal name foils were fabricated. Each stimulus was presented once, and subjects were asked to make a ‘yes/no’ judgement on familiarity.

#### 2.4.1 Results

Table 2 shows the results of familiarity judgments on voices, faces and names, in QR, KL and controls. For controls, the voice familiarity task was most difficult (mean score equivalent to 85% correct), compared to near-ceiling performance on face and name familiarity (mean score equivalent to 97% correct in each modality). QR performed close to chance (and significantly worse than controls: *t* = −3.8, *p* < 0.01, df = 22) for voice familiarity judgments; for face familiarity judgments, QR's performance was above chance but also significantly worse than controls (*t* = −10.8, *p* < 0.001, df = 22), while for name familiarity judgments her performance was significantly worse than the control mean (*t* = −2.2, *p* = 0.04, df = 22) but within the control range. Further analysis of errors made by QR revealed that she correctly classified only 15/24 familiar voices as familiar, and misclassified 14/24 unfamiliar voices as familiar. On name familiarity she correctly classified 19/24 familiar names as familiar and misclassified 0/24, while she correctly classified 19/24 familiar faces as familiar, but misclassified 14/24 unfamiliar faces as familiar (i.e., she showed an inflated false alarm rate, especially for face familiarity: 14/19 errors). KL's performance was significantly worse than controls for all three modalities (voices: *t* = −3.1, *p* < 0.01, df = 22, faces: *t* = −9.6, *p* < 0.001, df = 22, names: *t* = −8.3, *p* < 0.001, df = 22). Analysis of KL's errors revealed a hit rate of only 6/24 familiar voices, 11/24 familiar faces and 14/24 familiar names. He made few false alarms: only 2/24 unfamiliar voices, 4/24 unfamiliar faces and 5/24 unfamiliar names were classed as familiar.

#### 2.4.2 Comment

QR performed close to chance for voice familiarity judgments; in addition, her ability to judge the familiarity of faces was also clearly impaired, whereas her ability to judge the familiarity of names was somewhat less impaired. This pattern suggests some modality specificity to QR's person familiarity deficit. KL performed similarly whether judging the familiarity of public figures from voice, face or name, supporting a multimodal person familiarity deficit.

### 2.5 Experiment 2: identification of voices and faces

The ability to name or otherwise demonstrate recognition of voices and faces was assessed using the 24 public figures selected for the familiarity task. Subjects were asked to identify the voice or face as precisely as they could; the criterion for correct recognition was name (surname) or other identifying feature (e.g., an event closely associated with the person, occupational information), in line with the criteria used by Snowden et al. (2004). Controls were required to supply more specific contextual information. For voice stimuli, national or regional origin was not accepted, as this could be based on accent cues alone. As both patients had evidence for generalised word retrieval impairment on a standard naming task (Table 1) a cross-modal matching task was designed to maximise the opportunity to demonstrate recognition of voices and faces using an alternative procedure that did not rely on proper name retrieval. For both face and voice targets, three foil arrays were selected from the complete 24 item set. One array contained the 6 females from the complete set, a second array contained the 9 male politicians, and the third contained the 9 male media figures. Arrays were based on the individual's career, as this is likely to be an important organisational principle in the domain of person knowledge (Crutch & Warrington, 2004). Famous faces (with name foils only) were presented first, followed by famous voices (with faces and name foils presented simultaneously).

#### 2.5.1 Results

Table 2 shows the results of identification tasks for voices and faces in QR, KL and controls. Controls performed significantly better on tests assessing identification of faces than voices (naming: *t* = 5.9, *p* < 0.001, df = 21; recognition: *t* = 6.1, *p* < 0.001, df = 21); face recognition test performance was near-ceiling. Both QR and KL performed at floor and significantly worse than controls for both naming (*t* = −3.7, *p* < 0.01, df = 21) and recognition (*t* = −4.7, *p* < 0.001, df = 21) of famous voices. Both patients performed significantly worse than controls for face naming (QR: *t* = −5.6, *p* < 0.001, df = 22, KL: *t* = −6.7, *p* < 0.001, df = 22) and face recognition (QR: *t* = −8.1, *p* < 0.001, df = 22, KL: *t* = −24.0, *p* < 0.001, df = 22), however QR's performance improved substantially for recognition of faces compared with voices, and her performance was significantly superior to KL's (*χ*^2^ = 14.31, *p* < 0.001, df = 1).

On cross-modal matching tasks, control performance was near-ceiling for both voices and faces. For cross-modal matching of faces to names, both QR and KL performed significantly worse than controls (*t* < −400, *p* < 0.001, df = 19) but performed clearly above chance; QR's performance was significantly better than KL's (*χ*^2^ = 5.89, *p* < 0.05, df = 1). For cross-modal matching of voices to faces and names, both patients performed at chance and significantly worse than controls (*t* = −22.2, *p* < 0.001, df = 19).

The experimental control group here had a high average NART IQ (120.9, SD = 6.3) and a greater mean number of years of education than QR, raising the possibility that a generic factor such as IQ contributed to her voice recognition deficit. We do not have a premorbid estimate of QR's IQ, and any estimation based, for example, on demographic factors such as occupation would need to be made with caution in the individual case. Moreover, regression analysis in a larger control sample of older adults (*n* = 48) (Appendix B), showed no evidence of association between number of years of education or NART IQ and voice recognition performance. In order to further explore any IQ-related contribution to QR's voice recognition difficulty, we compared her performance on the voice recognition tasks with five healthy control subjects (3 females, 2 males) who had an average IQ typical for the greater London population (mean IQ 107.6, SD 6.7, range: 96–112). This control group included three controls from the experimental control group with lower IQs (mean IQ 110.3, SD 2.1, range: 108–112) and two additional older adult controls (IQs 96 and 111) not included in the main study as they did not complete the perceptual voice tests. QR's performance was significantly inferior to this lower-IQ control subgroup (*p* < 0.001) on the voice familiarity (*t* = −6.7, *p* < 0.001, df = 4), naming (*t* = −5.0, *p* < 0.001, df = 4) and recognition (*t* = −6.0, *p* < 0.001, df = 4) tasks.

#### 2.5.2 Comment

These findings corroborate the results of Experiment 1. QR had a severe impairment of voice identification, evident across the recognition and cross-modal matching procedures used here. Her ability to retrieve proper names from voice or face was clearly impaired, as anticipated on the basis of her general word retrieval impairment (Table 1). However, her ability to identify the same public figures from face information in the recognition and cross-modal matching conditions (which do not rely on naming), though deficient to healthy controls, was clearly superior to her ability to identify voices, and superior to KL's performance in either modality. QR's score on the voice recognition task was also highly significantly worse than the lower-IQ control group: it therefore seems unlikely that her voice recognition deficit was due to IQ factors. In line with previous work, control voice recognition scores were significantly lower than face recognition scores (Hanley et al., 1998). In the pilot control regression analysis (Appendix B), increased news exposure was positively associated with voice recognition score. It is unlikely this factor explains QR's voice recognition deficit, as QR rated in the highest category for the number of times per week she read or watched the news (Appendix A). QR's relatively good performance on face recognition appears initially somewhat paradoxical in relation to her poor performance on the face familiarity judgment: however, this pattern is likely to reflect an inflated false alarm rate (14/19 errors) on the face familiarity task.

### 2.6 Experiment 3: comparison with recognition of lower frequency faces

Quantifying any face recognition deficit in Experiment 2 was confounded by near-ceiling control performance on both face recognition and cross-modal matching tasks; furthermore, all the public figures selected were recognised better from face than from voice by controls, and face recognition performance may have been primed by previous presentation of the corresponding voices. We therefore selected from the pilot study stimuli an alternative set of 24 faces that were matched in accuracy of recognition and naming by controls to the voices used in the main study (recognition achieved by 77% of controls; mean = 76.7, SD = 8.7, range: 58–85%) (see Appendix C). Recognition by pilot study group controls was not significantly different between this set of faces and the 24 voices (Wilcoxon rank-sum Test: *z* = −1.1, *p* > 0.26). This alternative set of faces was administered to QR and KL.

#### 2.6.1 Results

On recognition of difficulty-matched faces (Table 2), QR's performance did not differ significantly from healthy controls for either face naming (*t* = −1.2, *p* = 0.26, df = 24) or recognition (*t* = −1.1, *p* = 0.30, df = 24). KL's performance remained significantly inferior to controls (naming: *t* = −2.8, *p* < 0.05, df = 24; recognition: *t* = −3.2, *p* < 0.001, df = 24).

#### 2.6.2 Comment

These findings support the concept of a relatively modality-specific deficit of voice recognition in QR, in contrast to the multimodal deficit of person recognition exhibited by KL.

### 2.7 Experiment 4: perceptual analysis of voices: vocal size and gender

QR's voice recognition deficit could in principle reflect impaired perceptual processing of voices. We designed a series of experiments to investigate this possibility. Perceptual analysis of voices was assessed in both QR and KL using tasks requiring encoding of the basic voice properties of vocal tract length and gender.

Perceptual judgment of speaker size is a fundamental task of auditory cognition in humans and other species, and vocal tract length (VTL) is an important cue for perception of speaker size by normal subjects (Ives, Smith, & Patterson, 2005). Here we assessed categorical (‘big’ versus ‘small’) judgements of vocal size based on a VTL cue. Stimuli in this test were based on sixteen consonant-vowel syllables recorded by a single male speaker and digitally resynthesised using a previously described algorithm (Kawahara & Irino, 2004) that allows apparent vocal tract length to be varied independently of glottal pulse rate (voice pitch). Each syllable was presented at two extreme VTL values, one corresponding to a speaker height of 2 m (equivalent to a very tall man, ‘big’) and the other to a height of 0.5 m (equivalent to a child, ‘small’), forming the basis for 32 trials (16 big and 16 small). Each syllable was randomly assigned (independently of VTL) one of four similar pitch values within the normal human male vocal range (116, 120, 138, 158 Hz). Examples of stimuli are available from the authors. On each trial, subjects heard a sequence of three repetitions of the identical syllable, and were asked to decide if the sounds were made by a big person or a small person.

Voice gender can be determined using various low-level perceptual features including pitch and VTL. In this test we sought to determine whether such low-level cues could be used to assign a gender label to the stimulus. 24 stimuli (12 males, 12 females) were selected from the test set for the voice familiarity task. On each trial the subject was asked to decide if the voice was male or female.

#### 2.7.1 Results

Table 3 shows the results of perceptual analysis tasks for voices and faces in QR, KL and controls. Both patients were able to judge gender and speaker size, and their performance was not significantly different to healthy controls (QR: *t* = 0.0, *p* = 0.97, df = 20; KL: *t* = −0.8, *p* = 0.44, df = 20).

#### 2.7.2 Comment

QR's satisfactory performance on these tests makes it unlikely that her impaired ability to identify voices was grounded in an early vocal perceptual deficit.

### 2.8 Experiment 5: discrimination of unfamiliar voices

Beyond early perceptual encoding but prior to the attribution of meaning it is likely that voice processing entails an interposed stage of representation of the voice as a complex auditory object (Griffiths & Warren, 2004; Warren, Scott, Price, & Griffiths, 2006). This apperceptive stage of vocal processing can be assessed by tasks requiring discrimination of unfamiliar speakers. We created a novel speaker discrimination task in which subjects were required to detect a change in speaker within spoken phrases (highly over-learned sequences comprising days of the week ‘Monday, Tuesday, Wednesday, Thursday’ or months of the year ‘January, February, March, April’). In order to control for gender, age and accent, all speakers were female, aged 21–31 years, with a standard Southern English accent. Inter-speaker variations in vocal pitch were controlled by fixing f0 of recorded stimuli at 220 Hz using Goldwave^®^ software. Recorded single words were concatenated with fixed inter-word gaps (0.1 s) to equate overall speech rate. Examples of stimuli are available from the authors. 24 trials were presented using spoken sequences of days of the week, followed by 24 trials using sequences of months. On each trial, subjects were asked to decide whether the spoken phrase contained a change in speaker (on change trials the change always occurred at the midpoint of the phrase, to maximise available vocal information for each speaker). Patient performance on these vocal tasks was compared with performance on a standard test of perceptual processing of face identity, the Benton Facial Recognition Test (Benton, Hamsher, Varney, & Spreen, 1989): this test depends on successful perceptual encoding of the configuration of a face, and requires the subject to match a photograph of target face to one (or three) of six other photographs of the target with distractor faces under different viewing conditions.

#### 2.8.1 Results

On the speaker discrimination task, QR's performance did not differ significantly from controls (*t* = 1.3, *p* = 0.22, df = 20) (Table 3). KL's performance was also not significantly different from controls (sample: *t* = −0.6, *p* = 0.54, df = 20). Both QR and KL performed normally on the Benton test of perceptual matching of faces.

#### 2.8.2 Comment

This experiment provides further evidence that pre-semantic vocal processing mechanisms were intact in QR and KL. Both patients were able to achieve an intact representation of individual voices as auditory objects sufficient to discriminate between different speakers, yet were unable to gain a sense of familiarity to a voice or to associate these representations with other stored information about familiar speakers.

### 2.9 Experiment 6: recognition of vocal emotions

Vocal emotion and identity information are likely to be at least partly dissociable cognitively and anatomically (Belin et al., 2004). Patients with FTLD (in particular, the so-called behavioural variant) often show altered responses to emotions in various input modalities, including voice (Keane, Calder, Hodges, & Young, 2002; Snowden et al., 2008). Processing of vocal emotion by QR and KL was therefore assessed in a separate experiment. 40 nonverbal vocalisations, 10 representing each of the emotions happiness, sadness, anger and fear, were selected from a previously developed set (Sauter & Scott, 2007; Sauter, Calder, Eisner, & Scott, in press). Items most reliably recognised by young normal subjects based on these previous normative data were selected. The subject's task on each trial was to select the emotion label describing the target emotion in a four-alternative forced choice format.

#### 2.9.1 Results

Table 3 shows the results of the vocal emotion recognition test for QR, KL and controls. Both QR and KL performed comparably to healthy controls (QR: *t* = −1.0, *p* = 0.34, df = 21, KL: *t* = −1.6, *p* = 0.12, df = 21).

#### 2.9.2 Comment

Considered together with the results of Experiments 1–3, these findings provide support for a dissociation between vocal identity and vocal emotion processing in these patients.

### 2.10 Experiment 7: identification of environmental sounds

It is not established to what extent the processing of voices is separable from other complex nonverbal sounds. We addressed this issue in a further experiment probing recognition of environmental sounds. 40 common environmental sounds representing a variety of sound sources, including elemental sounds (e.g., thunder), man-made objects (e.g., kettle whistling), and animal calls (e.g., cow mooing), were selected from on-line databases. Environmental sounds were identified either by sound source (e.g., cow or a tap), or a description of the sound (e.g., mooing or dripping water); relatively lenient criteria for recognition were used, in line with the criteria used for person identification (Experiments 2 and 3). In a cross-modal version of the test, the subject was presented with arrays of four names and pictures, and required to match each sound with the correct name-picture combination.

#### 2.10.1 Results

Table 4 shows the results of environmental sounds identification tests for QR, KL and controls. On the sound recognition test, both QR and KL performed comparably to healthy controls (QR: *t* = −1.0, *p* = 0.35, df = 14; KL: *t* = −1.4, *p* = 0.18, df = 14). On the cross-modal matching task, KL performed at ceiling and QR near-ceiling; 9/10 control subjects performed at ceiling on this task.

#### 2.10.2 Comment

Both QR and KL performed essentially normally on tests of environmental sound recognition. These findings suggest that the deficit of voice recognition exhibited by each patient is at least relatively specific for human voices.

### 2.11 Experiment 8: identification of musical instruments

Voice identification requires fine-grained perceptual and semantic processing within a single highly differentiated category of complex sounds. It is unclear therefore whether selective deficits of voice processing versus other kinds of complex sounds reflect the privileged ecological status of human voices or rather the greater demands of processing unique auditory exemplars. Similar arguments have previously been advanced to challenge claims that human faces constitute a privileged category of visual objects (Gainotti et al., 2008). Here we addressed this issue using an alternative finely differentiated category of complex sounds: musical instruments. Subjects were asked firstly to name 20 different sequentially presented instruments from their sounds (audio clips between 4 and 10 s in duration), and then to identify the same instruments in a cross-modal matching condition, in which instrument sounds were presented together with arrays of four written instrument names and pictures. Cross-modal arrays contained the target instrument, a within-instrument family distractor (e.g., woodwind, brass, strings, percussion, and keyboard), and two instruments from a different instrument family. As QR had no musical training and KL had only 2 years of childhood piano lessons, patient performance was compared to 12 controls with up to 2 years musical training (defined as “inexperienced listeners”: (Halpern, Bartlett, & Dowling, 1995)).

#### 2.11.1 Results

Table 4 shows the results of musical instrument identification tests for QR, KL and controls. Inexperienced listeners recognised on average 68.5% (SD = 14.4%) of the instruments, an accuracy level inferior to recognition of famous voices by the same controls. Both patients performed significantly worse than controls on tests of instrument sound naming (QR: *t* = −2.8, *p* < 0.05, df = 11; KL: *t* = −2.4, *p* < 0.05, df = 11) and recognition (QR: −2.6, *p* < 0.05, df = 11; KL: *t* = −2.2, *p* < 0.05, df = 11). On the cross-modal matching task QR performed above chance, however her score was significantly different to controls (*t* = −9.4, *p* < 0.001, df = 11); in contrast KL's performance was not significantly different to controls (*t* = −1.7, *p* = 0.12, df = 11). Both controls’ and patients’ performance improved on the visual version of the task. Both patients’ scores were significantly different to controls on tests of instrument picture naming (QR: *t* = −7.4, *p* < 0.001, df = 11; KL: *t* = −3.4, *p* < 0.01, df = 11) and recognition (QR: *t* = −4.7, *p* < 0.01, df = 11; KL: *t* = −2.8, *p* < 0.05, df = 11).

#### 2.11.2 Comment

These findings suggest that QR's ability to recognise another category of finely differentiated sounds (musical instruments) was impaired, though superior to her ability to recognise voices. In contrast, KL exhibited normal auditory recognition of instruments on the cross-modal matching task. This pattern of results might signify that QR has an auditory agnosia that affects recognition of voices and certain other categories of auditory objects, whereas KL has a primary deficit of person knowledge. However, this interpretation requires some qualification, since both patients also exhibited impaired visual recognition of instruments relative to the healthy control group, while QR scored lower on both the auditory and pictorial versions of the task relative to KL. It is difficult to equate musical exposure between non-musicians (KL's musical experience is likely to have been wider than QR's) and this may also be affected by other factors, such as general educational attainment (QR had fewer years of formal education than KL). These factors are likely a priori to be relatively more important for music than person knowledge. Moreover, no other category of complex nonverbal sounds is truly comparable in diversity and familiarity to human voices (for practical purposes, a musically untrained subject is likely to be acquainted with perhaps twenty or thirty musical instruments, but potentially hundreds of individual human voices).

## 3 Discussion

Here we have presented neuropsychological evidence for distinctive deficits of voice recognition in two patients with focal neurodegenerative disorders. The first patient, QR, exhibited severe impairments of voice identification and familiarity judgments with preserved recognition of difficulty-matched faces and environmental sounds; recognition of another highly differentiated category of complex sounds (musical instruments), though impaired, was substantially better than recognition of voices. In contrast, patient KL exhibited severe impairments of both voice and face recognition, partly preserved recognition of musical instruments and essentially normal recognition of environmental sounds. Both patients demonstrated preserved ability to analyse perceptual properties of voices to the level of individual speaker discrimination and to recognise emotions in voices. The profiles of deficits exhibited by both QR and KL are summarised in Table 5. QR's deficit of voice processing could be characterised as a failure to associate familiar voices with other specific semantic information about the individual: associative phonagnosia. Further, this deficit is relatively selective for voices. KL's more uniform deficit of recognition across modalities (voices, faces and names) suggests a multimodal failure of person knowledge with associative phonagnosia as one component.

Detailed studies of phonagnosia are comparatively few (Garrido et al., 2009; Neuner & Schweinberger, 2000; Van Lancker & Canter, 1982; Van Lancker & Kreiman, 1987; Van Lancker et al., 1988, 1989) and neuropsychological investigations of voice recognition have generally been undertaken in patients presenting with acquired or developmental prosopagnosia (Gainotti et al., 2008; Gentileschi et al., 1999, 2001; von Kriegstein et al., 2006). Selective phonagnosia has recently been described on a developmental basis (Garrido et al., 2009): this individual had deficits of voice recognition and familiarity despite normal face recognition. Deficits in person knowledge are well described as a presentation of right temporal lobe degeneration: selective impairment of face recognition and multimodal impairment extending to recognition of voices and names have been described (Evans et al., 1995; Gainotti, 2007a; Gainotti et al., 2003, 2008; Gentileschi et al., 1999, 2001; Joubert et al., 2003; Snowden et al., 2004; Thompson et al., 2004). It has previously been shown (Snowden et al., 2004; Thompson et al., 2004) that patients with predominant right temporal lobe atrophy are in general more impaired for recognition of faces than names, whereas patients with predominant left temporal lobe atrophy exhibit the reverse pattern of deficits. Considered together with the neuroimaging findings in the present cases (Fig. 1), this evidence suggests that the anterior temporal lobes instantiate mechanisms for processing multiple aspects of person knowledge and the right temporal lobe may be implicated particularly for aspects of nonverbal person knowledge. Associated broader deficits of verbal or visual semantics have been documented in a number of cases, leading to the proposal that person knowledge is mediated by a distributed bi-temporal network with dedicated brain regions representing modality-specific information. A substrate of this kind would allow for modality-specific biases within a more widespread defect of person knowledge (Neuner & Schweinberger, 2000): cross- or multimodal knowledge could be mediated by close connections within a common network or by additional dedicated (anterior temporal) mechanisms (Snowden et al., 2004; Thompson et al., 2004). However, phonagnosia has not previously been emphasised as the leading feature of person knowledge breakdown in degenerative disease, and detailed anatomical correlates of this deficit remain to be established.

The present findings speak to current cognitive models of the organisation of person knowledge and particularly the processing of human voices. Models of voice processing (Belin et al., 2004; Ellis et al., 1997; Neuner & Schweinberger, 2000) have been heavily influenced by hierarchical models of face recognition such as that developed by Bruce and Young (1986). The Bruce and Young model posits separable pathways for processing faces, voices and names that operate partly in parallel and generate structural representations of person information at modality-specific recognition units. Recognition units are linked to cross-modal Person Identity Nodes (PINs) that are linked independently in turn to amodal stored semantic information about the person (e.g., profession, nationality, autobiographical details). A sense of familiarity for the person could arise prior to the stage of explicit identification (e.g., at the PIN: (Burton, Bruce, & Johnston, 1990; Burton et al., 1993)). The model also incorporates a further functional separation of pathways processing identity information and affective signals. Belin and colleagues (2004) have proposed a hierarchical model of voice recognition based on the Bruce and Young model. According to this model, voices first undergo acoustic analysis in common with other classes of complex sounds in the ascending auditory pathways to primary auditory cortex, followed by a stage of vocal structural encoding proposed to engage cortical areas in bilateral upper superior temporal sulcus, and subsequent stages of speaker identification and association with cross-modal and amodal knowledge about the speaker are mediated in the anterior temporal lobe and beyond. Within this formulation, the superior temporal sulcus might plausibly instantiate voice-specific recognition units, and anterior temporal cortex the PIN.

This model has received experimental support from neuropsychological and functional imaging studies (Belin et al., 2000, 2002; von Kriegstein, Eger, Kleinschmidt, & Giraud, 2003; von Kriegstein et al., 2005; Warren et al., 2006). However, modality-specific deficits of person knowledge (in judgements of familiarity and also retrieval of semantic information) present a potentially critical test of the model, and indeed models of the semantic system more broadly (Gainotti, 2007a; Lucchelli & Spinnler, 2008; Mahon, Anzellotti, Schwarzbach, Zampini, & Caramazza, 2009; Snowden et al., 2004; Thompson et al., 2004). The multimodal impairments displayed by KL here (and by most previously studied patients with progressive prosopagnosia) are consistent with a core defect affecting a multimodal store of knowledge about familiar people (Gainotti, 2007a; Gainotti et al., 2008; Gentileschi et al., 2001; Lucchelli & Spinnler, 2008), reflecting either damage to the stores proper or a disconnection from the PIN. However, QR exhibits a relatively selective associative deficit of voice recognition. Such a deficit could in principle arise at pre-semantic stages in the voice processing pathway: the demonstration of intact early vocal perceptual analysis and speaker discrimination in QR would be consistent with a dissociation of perceptual descriptions or voice recognition units from the PIN. A lesion at this processing stage might also account for loss of the sense of familiarity of voices. However, while voices are often analogised as ‘auditory faces’, the demands of perceptual analysis differ substantially between the auditory and visual modalities, and mechanisms for the perceptual analysis of voices remain poorly understood. Deriving a faithful neural representation of a voice is likely to depend on intact mechanisms for processing timbre, the spectrotemporal signature that defines a voice as an auditory object (Griffiths & Warren, 2004; Warren et al., 2006). Selectivity of voice recognition deficits could arise from an abnormal interaction between combinations of complex vocal properties such as timbre, articulation and prosody which distinguish an individual's voice (Perrachione & Wong, 2007; Remez, Fellowes, & Rubin, 1997; Schweinberger, 2001; Van Lancker, Kreiman, & Cummings, 1985), and subsequent stages of voice identity processing (it is of interest that KL reported some difficulty understanding unfamiliar accents). Interaction between perceptual and semantic mechanisms of voice processing would be in line with recent re-evaluations of models of person identification (Lucchelli & Spinnler, 2008), and may be particularly critical under non-standard listening conditions (e.g., identification of voices over the phone or when singing: (Benzagmout, Chaoui, & Duffau, 2008; Garrido et al., 2009).

A related issue is the specificity of agnosia for voices versus other kinds of complex sounds and versus unique entities (i.e., items associated with proper nouns: (Gainotti et al., 2008)) in sound or other modalities. This speaks to the more fundamental issue of the degree of specialisation of brain mechanisms for processing voices versus other kinds of ecologically relevant complex sounds (Belin et al., 2004). Both QR and KL were able to recognise environmental sounds successfully, arguing against a generalised auditory agnosia: this dissociation corroborates previous findings (Garrido et al., 2009; Neuner & Schweinberger, 2000; Peretz et al., 1994). QR and KL demonstrated comparably weak performance for recognition of London landmarks, but in both cases this was clearly superior to recognition of voices (and in the case of KL, also superior to recognition of faces). Furthermore, QR demonstrated a clear superiority for recognition of faces versus voices. Taken together, these observations argue that phonagnosia in these cases is unlikely simply to reflect a generic defect of fine-grained semantic attributions. Within the auditory modality, both QR and KL showed superior recognition of musical instruments compared with voices, however QR's performance was clearly inferior both to healthy controls and KL. Musical instruments are themselves likely to constitute a specialised category of complex sounds, but (unlike voices) cannot strictly be considered ‘unique entities’: nevertheless, the pattern of QR's results raises the possibility that her phonagnosia is part of a broader defect of differentiation amongst closely related auditory entities, which could in turn arise at the level of associative (semantic) processing or as a result of an abnormal interaction between perceptual and semantic mechanisms. This formulation would be consistent with evidence in the visual domain, in both the present and previous studies e.g., (Gainotti, 2007a; Gainotti et al., 2008): patients with right temporal lobe lesions in general exhibit a more severe deficit for recognition of faces than landmarks and other unique visual entities, however this recognition deficit is seldom restricted purely to faces.

The present study shares the limitations of single neuropsychological case studies, including limited scope for anatomical correlation: this applies particularly to neurodegenerative pathologies, in which any regional selectivity of brain damage is relative rather than absolute. That caveat aside, these cases together illustrate a syndrome of progressive associative phonagnosia and demonstrate that this may be relatively selective with respect to other stages of voice analysis, other aspects of person knowledge and other categories of auditory objects. Important directions for future work will include the longitudinal study of the evolution of phonagnosia in relation to other defects of person knowledge in patients with degenerative pathologies, a more detailed examination of the processing of other unique or finely differentiated auditory entities in phonagnosic individuals, and structural and functional anatomical substrates for the syndrome.

## Figures and Tables

**Table:** 

	Experiment 1: main study figures	Experiment 3: difficulty matched faces
1	Alan Bennett	Alan Titchmarsh
2	Ann Widdecombe	Anne Robinson
3	Bill Clinton	Anthony Hopkins
4	Billy Connolly	Barbara Windsor
5	Bob Geldof	Bruce Forsyth
6	David Attenborough	Charles Kennedy
7	Edward Heath	Cilla Black
8	George Bush	David Cameron
9	Gordon Brown	David Frost
10	Ian Paisley	David Jason
11	Janet Street-Porter	Dolly Parton
12	Joanna Lumley	Hugh Grant
13	John Humphreys	Jack Nicholson
14	John Major	Jeremy Clarkson
15	Jonathan Ross	Jimmy Carter
16	Judi Dench	John Cleese
17	Kenneth Williams	John Snow
18	Margaret Thatcher	Michael Caine
19	Neil Kinnock	Michael Portillo
20	Prince Charles (written name: Charles Windsor)	Moira Stewart
21	Princess Diana (written name: Diana Spencer)	Robbie Coltrane
22	Ronnie Corbett	Sean Connery
23	Terry Wogan	Stephen Fry
24	Tony Blair	Woody Allen

**Table 1:** Summary of patient and control performance on background neuropsychological assessment.

	QR score (percentile)	KL score (percentile)	Controls mean (SD) *n* = 23
General neuropsychological tests			
NART full scale IQ	86	113	120.9 (6.3)
MMSE (/30)	28	25	n/d
WAIS III Digit Span (forwards, backwards)	12, 5 (60–80th)	14, 5 (80–95th)	n/d
Graded Naming Test (/30)	4 (<5th)	6 (<5th)	26.0 (2.0)
Synonyms—concrete (/25)	17 (10th)^b^	21 (50th)^b^	24.5 (0.7)
Synonyms—abstract (/25)	12 (1–5th)^b^	24 (75–90th)^b^	24.3 (0.8)
Object Decision task (/20)	19 (75–90th)	18 (50–75th)	17.8 (1.9)
D-KEFS Design Fluency task: switching	1 (<5th)	6 (50–75th)	n/d

Identification of unique visual entities			*n* = 17
Famous faces test: naming (/12)	4 (5th)	1 (<5th)	9.9 (1.7)
Famous faces test: recognition (/12)	8 (10–25th)	1 (<5th)	10.8 (1.2)
Landmark naming (/15)	7 (<5th)^a^	6 (<5th)^a^	13.6 (1.7)
Landmark recognition (/15)	8 (5th)^a^	8 (5th)^a^	13.7 (1.4)

Percentiles calculated from standardised tests, except where marked: a, calculated from previous healthy control sample (*n* = 143); b, test administered with both visual and auditory presentation of words whereas the standardised percentiles are calculated for auditory presentation only. n/d = test not performed.

**Table 2:** Results of experimental tests of familiarity and identification of public figures from voice, face and name in patients and controls.

	QR	KL	Controls *n* = 20–24 mean (SD)	Control range min–max
Voice				
Voice familiarity (/48) (% correct)	25 (52%)	28 (58%)	40.6 (4.0)	29–46
Voice naming (/24)	0	0	16.7 (4.4)	7–23
Voice recognition (/24)	0	0	18.8 (3.9)	10–23
Cross-modal matching to face/name (/24)	3	3	23.5 (0.9)	21–24

Face				
Face familiarity (/48) (% correct)	29 (60%)	31 (64%)	46.7 (1.6)	43–48
Face naming (/24)	6	3	21.4 (2.7)	16–24
Face recognition (/24)	17	4	23.6 (0.8)	21–24
Cross-modal matching to name (/24)	19	11	24.0 (0.0)	24–24
Difficulty-matched faces: naming (/24)	6	1	14 (6.8)^a^	2–24
Difficulty-matched faces: recognition (/24)	13	1	19 (5.6)^a^	3–24

Name				
Name familiarity (/48) (% correct)	43 (90%)	33 (69%)	46.6 (1.6)	42–48

a Pilot control sample (*n* = 26) scores for identification of 24 faces (see Appendix B) were used to assess performance on this test.

**Table 3:** Results of experimental tests of perceptual processing of voices and faces, and recognition of vocal emotion in patients and controls.

	QR	KL	Controls *n* = 21 mean (SD)	Control range min–max
Voice perception				
Gender discrimination (/24)	24	24	n/a	n/a
Size discrimination (/32)	29	25	28.8 (4.7)	17–32
Speaker discrimination (/48)	39	33	35.0 (3.1)	29–41

Face perception				
Benton facial recognition test (/56)	48	41	n/a	n/a

Vocal emotion recognition				
Cross-modal matching to emotion (/40)	32	30	35.1 (3.1)^a^	26–39

n/a = test not performed.

a Separate control group results (*n* = 22), were used to assess performance on this test.

**Table 4:** Results of experimental tests of environmental sound and musical instrument identification in patients and controls.

	QR	KL	Controls mean (SD) *n* = 12	Control range min–max
Environmental sounds				
Environmental sound recognition (/40)	35	34	37.1 (2.1)^a^	33–39
Cross-modal matching to picture/name (/40)	39	40	39.9 (0.3)^b^	39–40

Musical instruments				
Instrument sound name (/20)	5	6	13.1 (2.8)	8–18
Instrument sound recognition (/20)	6	7	13.7 (2.9)	9–18
Instrument picture name (/20)	4	11	17.1 (1.7)	14–19
Instrument picture recognition (/20)	10	13	17.3 (1.5)	15–19
Cross-modal matching sound to picture/name (/20)	12	18	19.3 (0.8)	18–20

a *n* = 14 controls.

b *n* = 10 controls.

**Table 5:** Summary of experimental neuropsychological profiles in QR and KL.

	Domain	Case QR	Case KL
Voices	Identification	↓	↓
	Familiarity	↓	↓
	Emotion recognition	N	N
	Perception	N	N

Other sounds	Musical instrument matching	↓	N
	Environmental sound recognition	N	N

Faces	Recognition	N^a^	↓↓
	Perception	N	N

N: normal performance, **↓**: impaired performance relative to controls, **↓↓**: impaired performance relative to both controls and other case.

a When matched to voices for difficulty.

## Appendix A

Subjects’ exposure to the media.

All subjects completed a questionnaire detailing their background media exposure; subjects were asked to estimate their average media exposure for the preceding three months according to one of three categories: number of hours per week spent watching television, number of hours per week spent listening to the radio, and number of times per week they watched or read the news. QR's media exposure was greater than the average experience of the controls in each of these categories: on average each week she spent over 20 h watching television (median control category = 5–10 h per week, range: 0–20+), over 20 h listening to the radio (median control category = 5–10 h per week, range: 0–20+), and read or watched the news more than 10 times (median control category = 8–10 times per week, range: 0–10+). In contrast KL reported lower than average exposure in all categories but fell within the control range. He currently listened to the radio 0–1 h per week (a change from 1–5 h per week prior to symptom onset), he read the news once a week, and did not regularly watch television.

## Appendix B

Quantification of voice recognition ability: control pilot study.

A pilot control sample of 26 older controls, 18 females (mean age = 65.5, SD = 7.3, range: 51–82) were tested using voices and face stimuli, obtained from publicly available sources. Familiarity, naming and recognition of 60 famous and 60 unfamiliar voices and faces were assessed, presenting the same public figures in both modalities. Order of presentation of faces and voices was balanced between subjects. One control subject from the pilot study was excluded, based on performance greater than 2 standard deviations below the control mean on all tests of voice and face identification; this may reflect a developmental or acquired difficulty with person recognition.

To select items for the final study, the same public figures were used in three modalities to directly compare knowledge of representations from voice, face and name. As the voice is the modality of interest, the 24 public figures best recognised from voice by pilot study controls were selected; these items were all recognised by 60–100% of controls and for consistency across items, all voices selected were recognised more easily from face than from voice. A selection of unfamiliar voices and faces that were classified as unfamiliar by >75% of controls were also selected for the final test.

The pilot control sample scores for naming and recognition of the 24 voices and faces used in the final study were combined with scores from the main study control sample. Statistics for the total control sample of 48 controls, 33 females (mean age = 64.8, SD = 5.9, range: 51–82) were calculated. Voice identification scores (naming: mean = 16.2, SD = 4.9, range: 5–23; recognition: mean = 18.4, SD = 4.6, range: 5–24) were lower than scores for identification of the faces (naming: mean = 20.1, SD = 3.8, range: 8–24; recognition: mean = 22.8, SD = 2.0, range: 15–24). Paired *t*-tests were used to assess the difference in control scores between face identification and voice identification scores. Face naming scores were significantly greater than voice naming scores (*t* = 7.1, *p* < 0.001, df = 47) and face recognition scores were significantly greater than voice recognition scores (*t* = 7.9, *p* < 0.001, df = 47).

In order to assess the relationship between background control variables and voice and face identification test scores, we tested univariate associations between the effects of age, sex, number of years of education, IQ (measured on the NART), naming ability (assessed on the GNT), and media exposure categorical data (see Appendix A): for television watching (hours per week), radio listening (hours per week) and news exposure (see Appendix A) on famous voice naming, voice recognition, face naming and face recognition test scores, respectively. To allow for violations of the normality assumption, bias-corrected bootstrapped confidence intervals (CI) were used.

There was statistically significant evidence that an increase in exposure to the news was associated with an increase in voice recognition score (coefficient = 1.42 (CI 0.21, 2.86)). An increase in one news exposure category (categorised by the number of times a subject read or watched the news per week) resulted in 1.42 more public figures recognised by voice. There was no evidence of an association between voice recognition and any other background variables (age, sex, number of years of education, NART IQ, GNT score, television watching or radio listening). An increase in naming score on the GNT was significantly associated with an increase in voice naming score (coefficient = 0.58 (CI 0.11, 1.11)), an increase in face naming score (coefficient = 0.74 (CI 0.42, 1.10)) and an increase in face recognition score (coefficient = 0.22 (CI 0.04, 0.44)), respectively. An increase in age was statistically significantly associated with a decrease in: voice naming score (coefficient = −0.30 (CI −0.55, −0.08)), face naming score (coefficient = −0.30 (CI −0.50, −0.13)) and face recognition score (coefficient = −0.08 (CI −0.19, −0.01)). There was no evidence of association between any other background control variable (sex, number of years of education, NART IQ, TV watching or radio listening) and voice naming, face recognition or face naming scores.

## Appendix C

Lists of the public figures selected for Experiments 1 and 2 and faces difficulty matched to voices in Experiment 3.
